# Production of Smoked Sausage Using *Monascus* Pigments-Calcium Carbonate Colorant Lake with Nisin as a Nitrite Substitute

**DOI:** 10.3390/foods14030477

**Published:** 2025-02-02

**Authors:** Jiaqi Cui, Guohui Bai, Yifen Fu, Xu Zhai, Le Jing, Yuhan Liu, Dongdong Yuan, Chengtao Wang

**Affiliations:** Beijing Engineering and Technology Research Center of Food Additives, Beijing Technology & Business University, Beijing 100048, China; c18647362147@163.com (J.C.); xbxhzznc@163.com (G.B.); 18834695836@163.com (Y.F.); zhaixu12356@163.com (X.Z.); jingle1998@163.com (L.J.); liuyuhan_210@163.com (Y.L.); wct5566@163.com (C.W.)

**Keywords:** *Monascus* red, colorant lake, nisin, nitrite, smoked sausage

## Abstract

This study explored the complete replacement of sodium nitrite with a combination of *Monascus* pigments (MPs)-calcium carbonate colorant lake (MPs-CaCO_3_ lake) and nisin in smoked sausage production. The effects of the replacement on color stability, total aerobic mesophilic bacteria count (TAMB), and physicochemical properties of sausages were assessed. The results indicated that combining 0.26 g/kg of lake and 0.4 g/kg of nisin effectively replaced the coloring and preservative functions of nitrite. Physicochemical analyses revealed that the addition of pigment lake significantly increased the pH and calcium content and reduced juice loss rates (at low lake concentrations) of sausage in the lake group compared to the blank and pigment groups. Gas chromatography–mass spectrometry (GC-MS) based flavor compounds analysis demonstrated notable changes in the profile of volatile flavor compounds with the addition of MPs, marked by the appearance of paraldehyde and the disappearance of butanediol in the pigment and lake groups. Electronic nose analysis confirmed that sausages with MPs and lake had similar odors, distinctly different from the blank group. However, electronic tongue analysis showed no significant flavor differences among the three groups. Overall, the combination of MPs-CaCO_3_ lake and nisin effectively replaced nitrite, enhanced pigment stability, and did not adversely affect the flavor quality of smoked sausage.

## 1. Introduction

As a food additive, nitrite significantly contributes to the safety and sensory qualities of meat products. It effectively inhibits the proliferation of detrimental microorganisms, forestalls lipid oxidation and spoilage, and imparts a desirable pink hue and distinctive aroma to the meat product [1]. With the growing public awareness of health and nutrition, the potential adverse effects of nitrites have come under scrutiny. Elevated consumption of nitrites has been linked to an increased risk of developing bladder and stomach cancers [2]. Furthermore, excessive nitrite intake may elevate methemoglobin levels in infants, potentially resulting in methemoglobinemia. Commencing in the late 20th century, the pursuit of substitutes for nitrites in meat products has emerged as a crucial strategy aimed at diminishing the formation of nitrosamines in foods, a recognized health risk, given the growing scrutiny over the use of nitrites in foods. Currently, many studies have shown that it is possible to use active substances from plants, which act as antioxidants and antibacterials, among other things, as an alternative to nitrites [3]. For example, lycopene and polyphenols in dragon fruit have antioxidant, antibacterial and other effects and can be used in meat instead of nitrites [4]. Black tea has antioxidant properties and has been successfully used as a substitute for nitrites [5]. In addition to the active ingredients in plants, some microorganisms such as coagulase-negative staphylococci [6] and *Lactobacillus plantarum* [7] can have a nitrite-substituting effect. Nisin has strong antimicrobial activity against most Gram-positive food-borne pathogens and some Gram-negative pathogens [8]. Moreover, when acting together with other antibacterial substances, its antibacterial effect is enhanced [9]. However, these substitutes typically replicate only a subset of nitrite functions, and a comprehensive replacement for nitrites remains elusive.

*Monascus* pigments (MPs), are secondary metabolites produced by *Monascus* spp. and are natural occurring colorants. Natural MPs are invariably composed of a blend of colorants, with six primary compounds identified to date. These include monascorubramine and rubropunctamine, which impart a red color; monascorubrin and rubropunctain, responsible for orange tones; and ankaflavin and monascin, which provide a yellow color [10]. MPs have other biological activities such as antioxidant, antitumor and antibacterial activities [11]. The red pigments derived from MPs have been traditionally employed as a natural colorant in a myriad of food products, including an array of processed meats, fish, pickled sausages, pickled tofu, and red rice wine [12]. Beyond its culinary uses, the red MPs serve multifaceted roles as a preservative, a flavor enhancer, and a functional food additive [13]. Their utility extends to a broad spectrum of sectors such as food, pharmaceuticals, textile, dyeing, cosmetics, and more [14]. MPs have been used as a coloring agent for meat and have a coloring effect similar to that of nitrite [15]. They can be used as an alternative to sodium nitrite for dyeing effects. Moreover, it has been shown that the antioxidant, antibacterial, coloring and organoleptic properties of MPs added to smoked sausages were not significantly different from those of smoked sausages to which only sodium nitrite was added [16]. This indicates that MPs are good prospects for replacing sodium nitrite in meat products. However, as natural pigments, red MPs are susceptible to degradation and discoloration, influenced by diverse environmental pressures during the processing and storage phases [17]. The literature documents various strategies to enhance pigment stability. Notably, colorant lake technology stands out by offering multifaceted benefits such as increasing pigment stability [18], augmenting coloring capabilities [19], and expanding application scenarios of natural pigments [20]. This approach is recognized as the most efficacious among all examined technical solutions.

Aluminum hydroxide is traditionally used as a substrate for preparing colorant lake [21]. However, the presence of aluminum in infant foods poses potential neurotoxic risks to infants [22]. Aluminum is also difficult to eliminate from the human body, leading to its accumulation in critical organs such as the lungs, liver, kidneys, thyroid gland, and brain [23]. Furthermore, aluminum exposure is associated with oxidative stress and mitochondrial dysfunction, which can accelerate apoptosis [24]. Our team has recently developed an innovative colorant lake preparation technology that utilizes calcium carbonate as a substrate [25]. This groundbreaking approach seeks to entirely substitute aluminum hydroxide with calcium carbonate, thereby aiming to minimize aluminum absorption from food sources. The preparation of calcium carbonate-based colorant lake is achieved through a coprecipitation method, wherein the majority of the pigment occupies the surface area of the particles in the colorant lake while a minor portion is integrated within the particles. This pigment-binding process is concurrently associated with calcium carbonate’s formation, crystallization, and subsequent crystal transformation, entailing simultaneous adsorption and desorption phenomena. Studies have shown that chemical binding, electrostatic attraction and physical entanglement are potential driving forces for the formation of calcium carbonate lake [26,27]. Beyond water-soluble pigments, we have also explored calcium carbonate-based lake with fat-soluble pigments [28]. Overall, the calcium carbonate lake technique satisfies the imperative to protect pigments, enhance stability, and augment dyeing ability, demonstrating promising application potential.

In this study, MPs-CaCO_3_ lake was applied with nisin into the production of smoked sausages to evaluate the potential of the combination (MPs-CaCO_3_ lake and nisin) in replacing nitrite. The evaluation also included a comparison between samples prepared with MPs-CaCO_3_ lake and that prepared with MPs to investigate the influence of lake on the quality, especially on the color of the sausages. In this study, the influence of MPs-CaCO_3_ lake on the redness of the sausages and the pigment’s stability during storage was firstly investigated. Then, the amount of colorant lake required in smoked sausage to replace nitrite was investigated, and the color and color stability of the colorant lake-added sausage were compared with those of the pigment-added sausage and the nitrite-added sausage to determine the application effect of the colorant lake in the smoked sausage. Finally, the physicochemical, organoleptic and flavor properties of the smoked sausages during the storage period were analyzed.

## 2. Materials and Methods

### 2.1. Materials and Chemicals

MPs (BS, water soluble) were purchased from Shanghai Yuanye Bio-Technology Co., Ltd., Shanghai, China. Calcium chloride (98% *w*/*w*), sodium carbonate (99.9% *w*/*w*), sodium hydroxide (99% *w*/*w*), nisin (≥1000 IU/mg) and sodium nitrite (98% *w*/*w*) were purchased from Shanghai Macklin Biochemical Co., Ltd., Shanghai, China, and plate counting agar (PCA) was purchased from Beijing MREDA Technology Co., Ltd., Beijing, China. Smoke liquid (Red Arrow, El Paso, TX, USA) and pork hind leg meat were purchased from Yonghui Supermarket in Beijing, China. Hydrochloric acid (37%) was purchased from Beijing Chemical Industry Group Co., Ltd., Beijing, China. All the solutions needed in the experiment were prepared with deionized water, which was produced by a laboratory water purification system (HYP-QX-UP, Huiyipu (Beijing) Environmental Protection Technology Co., Ltd., Beijing, China).

### 2.2. Preparation of Colorant Lakes

First, 2.1 g of calcium chloride was accurately weighed and dissolved in 25 mL of deionized water. Similarly, 2.0 g of sodium carbonate was weighed, added to 200 mL of deionized water, and magnetically stirred until fully dissolved. Subsequently, MPs were introduced into the sodium carbonate solution at a 6 mg/mL concentration. The solution’s pH was then adjusted to 10.5 using 1 M HCl/NaOH solutions. Following this, the prepared calcium chloride solution was incorporated into the pH-adjusted mixture solution comprising sodium carbonate and pigment. This combination was stirred continuously for 26 min using a magnetic stirrer, followed by centrifugation at 3000 rpm for 15 min. Finally, the resultant MPs-CaCO_3_ lake precipitate was dried in an oven at 45 °C for 12 h.

### 2.3. Preparation of Smoked Sausages

Fresh pork thigh meat was chosen as the main ingredient and cut into cubes before being mixed with pork fat at a 1:3 fat-to-lean meat ratio. The mixture was minced using a mincer (MJ-JD55, Midea Group, Foshan, China) in order to produce a meat stuffing. For each kilogram of stuffing, 32 g of refined salt, 70 g of sugar, 100 mL of soy sauce, 20 mL of white wine, and 2 g of monosodium glutamate were added. Sausages were grouped as follows: a blank group without additional ingredients; a nitrite group with 0.15 g/kg sodium nitrite; a MPs group with a certain amount of MPs and nisin; and a lake group with a certain amount of MPs lake and nisin. The same proportions were employed for all other ingredients in all sausage groups. All ingredients were dissolved or dispersed in water, comprising 17.5% of the stuffing’s weight, and then thoroughly mixed into the meat. The mixture was marinated for 30 min. It was then filled into the casing (pig small intestine casing) using a manual rotary enema device (xcs0540, Necooks, Huizhou, China) equipped with a 1.5 cm diameter enema tube, with each section tied at 12–15 cm intervals using hemp rope. Fine needles were used to prick holes, allowing air and moisture to escape. The sausages were steamed at 75–80 °C for an hour, ensuring that the center temperature of the sausages remained above 75 °C throughout the process. After cooking, they were immersed in a 1.5% (*v*/*v*) smoke liquid at 0–4 °C for 15 min, drained, and placed in an oven at 60–70 °C for 30 min to dry the casings. It should be noted that to minimize the complexity of the sausage system and reduce interference factors when investigating the effects of pigment and lake addition, the use of ingredients were minimized in the preparation of smoked sausages for this experiment.

#### 2.3.1. Substitution of Sodium Nitrite with MPs

MPs were added at varying concentrations during the preparation of smoked sausage. The redness of the sausages, post-smoking, were then determined at each pigment concentration. Subsequently, the quantity of MPs that would make the redness of sausages equal to that of the sausage with 0.15 g/kg of sodium nitrite (the maximum permissible amount) was calculated. The formulation details are shown in Table 1 (Samples 1–7).

#### 2.3.2. Substitution of Sodium Nitrite with MPs-CaCO_3_ Lake

Similar to Section 2.3.1, MPs-CaCO_3_ lake was added at varying concentrations during the preparation of smoked sausage to replace sodium nitrite. The redness of the sausages, post-smoking, was then determined for each lake concentration. Subsequently, the quantity of lake that would make the redness of the sausages equal to that of the sausage with 0.15 g/kg of sodium nitrite (the maximum permissible amount) was calculated. The formulation details are shown in Table 1 (Samples 8–12).

#### 2.3.3. Influence of Nisin Concentration on the Substitution of Sodium Nitrite with Colorant Lake

In this section of the experiments, nisin was added at varying concentrations with a constant concentration of MPs lake (0.26 g/kg) during the preparation of smoked sausage to replace sodium nitrite. The redness of the sausages, post-smoking, was measured at each nisin concentration. Sausages from the blank group and nitrite group were used as controls to investigate the effect of nisin content on the lower in total aerobic mesophilic bacteria (TAMB) during storage. The specific additions are detailed in Table 1 (Samples 13–17).

### 2.4. Characteristic Analysis of Smoked Sausages

#### 2.4.1. Redness

The color attributes of the produced sausage samples were assessed using a colorimeter (CM-3610A, KONICA MINOLTA, Tokyo, Japan). For this analysis, sausages were sliced and packaged in transparent vacuum compression bags. From each sausage, two slices were selected and three measurements were taken per slice to ascertain the L, a, and b values, where ‘L’ signifies brightness, ‘a’ represents the red (+) to green (−) spectrum, and ‘b’ indicates the yellow (+) to blue (−) spectrum. The index ‘a’ reflects the degree of redness of the sausages, and their change (Δa) indicates the stability of the red color.

#### 2.4.2. Color Stability

The variation in redness of the sausages (blank group, MPs group, and lake group) when subjected to light exposure and steaming treatment were measured to evaluate their color stability. For the light stability experiment, one sample was divided into two parts. The Δa value was calculated by subtracting the ‘a’ value of one part of the sample stored in a dark environment from that of another part stored under illumination. Measurements of the ‘a’ value were conducted every two days, and the testing period lasted for a total of 30 days. For the steaming stability experiment, the Δa value was calculated by subtracting the ‘a’ value after steaming from the ‘a’ value before steaming treatment. The steaming procedure was as described in Section 2.3.

#### 2.4.3. Total Aerobic Mesophilic Bacteria Count (TAMB) Analysis

The total aerobic mesophilic bacteria count (TAMB) of the sausages was determined following the protocol established by Huang, et al. [15]. A 5.0 g sample was accurately weighed and homogenized in a sterile bag containing 45 mL of saline for 1 to 2 min, creating a 1:10 homogenate (FB-110S high pressure stomacher, Shanghai Litu Ultra High Voltage Equipment Co., Ltd., Shanghai, China). Subsequently, 1 mL of this homogenate was taken using a 1 mL pipette and serially diluted to concentrations of 10^−4^, 10^−5^, and 10^−6^. Then, 1 mL of each dilution was spread onto sterile Petri dishes in triplicate. These plates were incubated at 36 °C for 48 h, after which the colonies were counted. Measurements of the value were conducted every three days, and the testing period lasted for a total of 30 days.

### 2.5. Physical and Chemical Indexes of Smoked Sausage

pH value: The pH values of sausage samples during storage were determined following the methodology described by Yang, et al. [29]. Samples were collected at the onset (day 0) and subsequently every four days across a 28-day storage period. Each time, a 10.0 g portion of a sausage was taken and placed into a sterile homogenizing bag, to which 90 mL of distilled water was added. The sample was homogenized by a high pressure stomacher (FB-110S, Litu, Shanghai, China), the resultant mixture was filtered, and the filtrate’s pH was measured with a pH meter (PHS-25, INESA, Shanghai, China).

Calcium content: Calcium content of smoked sausage was determined according to the method from Zhang, et al. [30] with slight modifications. About 5.0 g of a sausage sample after removing the casing was weighed into a quartz digestion tube, and then 4 mL HNO_3_ solution (65%, *v*/*v*) and 1 mL H_2_O_2_ solution (30%, *v*/*v*) were mixed into the tube, which was then subjected to microwave treatment (MWD-500, Metash instrument Company, Shanghai, China) for 1.5 h. The resulted digestion solution was diluted 100-fold with deionized water before measurement by inductively coupled plasma–mass spectrometry (ICP-MS) (Agilent 7500 series, Agilent Technologies, Santa Clara, CA, USA).

Juice loss: The juice loss ratio was determined by monitoring the weight change of the smoked sausages at 4-day intervals during storage. Before each measurement, the surface moisture was removed using absorbent paper. The juice loss of the sausages was calculated using Equation (1).

(1) $$Juice\;loss\left(\%\right)=\frac{Initial\;weight-Weight\;after\;storage}{Initial\;weight}\times 100\%$$

### 2.6. Flavor Analysis of Smoked Sausages

Solid-phase microextraction (SPME) sampling: the SPME fiber was preconditioned at the inlet of an Agilent 6890 gas chromatograph as per the manufacturer’s guidelines. Smoked sausage was finely chopped and ground, and 2.5 g of this prepared sample was transferred into a 15 mL vial. The vial was securely capped, sealed with film, and stored at 4 °C for the pre-analysis preservation of volatile flavor compounds. Prior to the actual analysis, the vial was acclimatized to room temperature for 1 h. It was then heated to 50 °C for 20 min on a headspace autosampler to achieve equilibrium, followed by the insertion of the SPME fiber for a 30 min adsorption period.

GC-MS conditions: Following adsorption, the samples underwent analysis via a GC-MS system (QP2010 Ultra, Shimadzu, Kyoto, Japan). The SPME fiber was thermally desorbed at the GC inlet at a temperature of 260 °C for a duration of 5 min. A split injection technique was employed with a ratio of 10:1. The separation of volatile compounds was achieved using an HP-1 capillary column (30 m × 250 µm, 1.0 µm). The temperature program for the column began at 38 °C, maintained for 2 min, followed by an increase to 100 °C at a rate of 3 °C/min, and finally to 240 °C at 10 °C/min, where it was held for an additional 2 min, totaling about 39 min. High-purity helium served as the carrier gas with a flow rate of 1.0 mL/min, and the interface temperature was set at 280 °C. The mass spectrometer, equipped with an electron multiplier detector and using electron impact ionization (70 eV), collected data in a mass range of 30 to 450 u. The mass spectral data was compared against the NIST 05 database for qualitative compound identification, ensuring a minimum match of 80%. Quantification of volatile compounds was based on peak area analysis.

Electronic nose: a quantity of 3.0 g of minced meat samples was hermetically sealed within a headspace vial and allowed to stabilize at room temperature for 1 h to equilibrate the volatile flavor compounds. Subsequent analysis was performed using an electronic nose (PEN3, Schwerin, Germany). The parameters for measurement included a sampling interval of 1 s, a pre-sampling duration of 5 s, a self-cleaning period of 100 s, a zeroing time of 10 s, an inlet flow rate of 300 mL/min, and a measurement duration of 90 s. The device was fitted with 10 distinct sensors, each attuned to specific odor compounds.

Electronic tongue: a sample of 25.0 g of minced meat was mixed with 125.0 mL of deionized water and placed in a water bath at 40 °C for 30 min. This was followed by low-speed stirring in a meat grinder for 1 min and centrifugation at 5000 r/min for 10 min at 4 °C. The supernatant was filtered, and the filtrate was analyzed using an electronic tongue (SA402B, Insent, Toyko, Japan). The instrument was equipped with five taste sensors for freshness, astringency, saltiness, sourness, and bitterness, as well as two reference electrodes.

### 2.7. Statistical Analysis

Microsoft Excel 2019 and Origin 2019 were used for data processing and graphing. The experiment prepared three distinct batches of sausages, and measurements were made in triplicate for each batch. A total of 200 sausages were produced and analyzed during the experiment. and the results are presented as mean ± standard deviation. Data were compared by ANOVA (IBM SPSS 24.0), and difference was regarded as significant when *p* < 0.05.

## 3. Results and Discussion

### 3.1. Substituting Sodium Nitrite with MPs

MPs have been reported to replace sodium nitrite in the production of meat products [11]. In this section, smoked sausages with MPs or sodium nitrite were produced and compared. The concentration of sodium nitrite was set as 0.15 g/kg, which is the highest concentration admitted in China according to the National Standard for Uses of Food Additives (GB 2760-2024 [31]).

**Figure 1:**
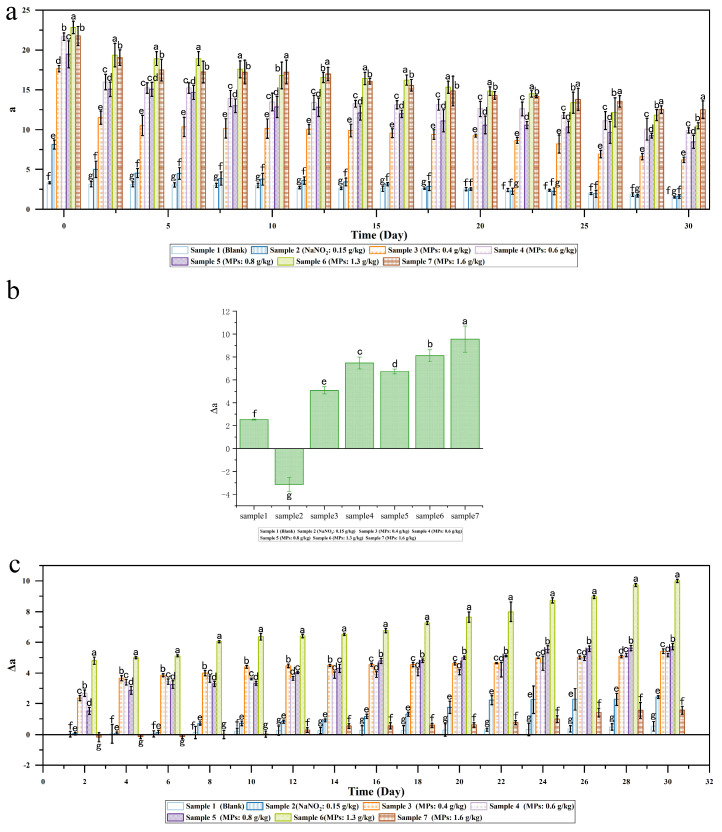
(**a**) Effect of MPs dosage on smoked sausage’s redness during storage; (**b**) Effect of MPs dosage on heat stability of smoked sausage’s redness; (**c**) Effect of MPs dosage on light stability of smoked sausage’s redness during storage. Different letters indicate statistically significant differences between the groups (*p* < 0.05).

Figure 1a illustrates the variation in redness (‘a’ value) of smoked sausage samples. Compared to the blank sample (Sample 1), the addition of sodium nitrite and MPs significantly enhanced the ‘a’ value of sausages (*p* < 0.05) throughout the experiment. The ‘a’ value of the blank sample (Sample 1) was significantly lower than that of the sodium nitrite-added sample (Sample 2) during the first 16 days of the storage period, after which they became nearly equal for the remainder of the storage period. All samples, except the blank, exhibited a general downward trend in ‘a’ value during storage. The blank sample’s ‘a’ value remained relatively stable for the first 25 days before ultimately decreasing by 54.7%. In contrast, the sodium nitrite-supplemented sample (Sample 2) experienced a more substantial decrease of 80.2%. Samples supplemented with MPs (Samples 3–7) showed varying degrees of color retention, with attenuation ratios of 64.9%, 54.3%, 56.5%, 54.0%, and 42.5%, respectively. These results suggest that the redness imparted by MPs is more stable than that provided by sodium nitrite. At the outset of the experiment, a comparison between Samples 3 and 4 revealed that the ‘a’ value was roughly proportional to the amount of MPs added. However, this proportional relationship did not hold for higher concentrations of MPs (Samples 5, 6, and 7). This phenomenon could be attributed to a shielding effect among adjacent MP molecules when pigments accumulate excessively. Based on the linear correlation between redness and MP concentration at lower levels, it was calculated that the redness of sausage with 0.15 g/kg sodium nitrite was approximately equivalent to that achieved with 0.24 g/kg MPs.

Figure 1b illustrates the stability of sausage’s redness under steaming treatment. The change in ‘a’ value after steaming is represented by Δa, with a higher Δa indicating more significant redness fading. Interestingly, Sample 2 (sodium nitrite-supplemented) exhibited a negative Δa, suggesting an enhancement of redness after steaming. This is due to the fact that the color of meat is determined by the concentration and redox state of myoglobin. When sodium nitrite is added to the meat, it reacts with myoglobin to form nitrosomyoglobin, which appears red. Following heating, nitrosomyoglobin is transformed into nitrosyl hemochrome, resulting in a pink color [32,33]. This process ultimately leads to a higher ‘a’ value after heating. All other samples displayed positive Δa values, indicating that steaming significantly reduced their redness. This effect was more pronounced in samples with higher concentrations of MPs (Samples 6 and 7). This aligns with previous research showing that heat treatment at various temperatures led to significant MPs degradation [34]. The blank sample (Sample 1) also showed a positive Δa, indicating the loss of redness color. This is because that heating treatment would denature the myoglobin, leading to the exposure of heme and increase the susceptibility of heme to oxidation, which finally causes the formation of brown ferrihemochrome [33]. In summary, the redness stability of MPs against steaming was inferior to that of sodium nitrite. This finding underscores the need to enhance MP stability to achieve effective substitution of sodium nitrite in sausage production.

The stability of the sausages’ redness against light exposure was tested as shown in Figure 1c. The ‘a’ values of the samples stored in dark and light exposure environment were monitored, and their difference was calculated to reflect the color stability of the corresponding samples. As shown in Figure 1c, for all of the samples, the Δa values increasingly rose during the test, confirming that light exposure accelerated the fading of redness. These results were consistent with the former reports in the literature, where the loss of color is due to the photo-degradation of *Monascus* pigments, primarily caused by the absorption of light energy by the chromophoric functional groups within the pigment molecules. The degradation mechanism involves electron transfer from the carbon–carbon double bond to the carbon–carbon single bond, leading to the breakdown of the pigment structure [35]. After exposure to light, the Δa values were all positive, and the Δa values of Sample 2 (sodium nitrite added at 0.15 g/kg) were all less than the Δa of the sausages with MPs, indicating that the red color caused by sodium nitrite had higher stability against light exposure than that derived from MPs.

In conclusion, these results demonstrate that sausages can achieve a level of redness equivalent to that produced by the maximum allowed concentration of sodium nitrite (0.15 g/kg) when supplemented with a relatively low concentration of MPs at 0.24 g/kg. This finding suggests the potential of MPs as a natural alternative to sodium nitrite for color enhancement in sausage production. However, the results also revealed significant limitations in the performance of MPs compared to sodium nitrite. Specifically, MPs exhibited inferior stability in maintaining redness when subjected to light exposure and heat treatment. These factors are crucial considerations in food processing and storage, potentially impacting the visual appeal and perceived quality of the final product over time.

### 3.2. Substituting Sodium Nitrite with MPs-CaCO_3_ Lake

Blank sausages and sausages with the addition of sodium nitrite or MPs-CaCO_3_ lake were prepared and compared in terms of their dyeing ability and color stability. Furthermore, the influence of MPs-CaCO_3_ lake on the redness of the sausages was investigated by varying the amount of added lake (with a loading capacity of MPs at 46.66%) as follows: 0.26 g/kg (containing 0.12 g/kg MPs), 0.36 g/kg (containing 0.17 g/kg MPs), 0.51 g/kg (containing 0.24 g/kg MPs), 0.67 g/kg (containing 0.31 g/kg MPs), and 0.77 g/kg (containing 0.36 g/kg MPs).

**Figure 2:**
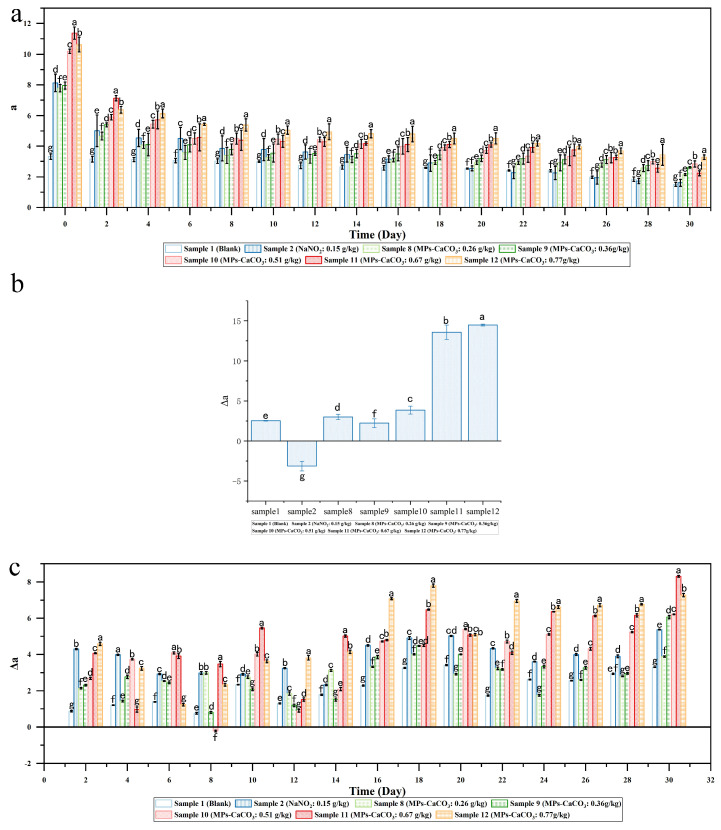
(**a**) Effects of lake dosage on smoked sausage’s redness during storage; (**b**) Effect of lake dosage on heat stability of smoked sausage’s redness; (**c**) Effect of lake dosage on light stability of smoked sausage’s redness during storage. Different letters indicate statistically significant differences between the groups (*p* < 0.05).

As illustrated in Figure 2a, throughout the experiment, all samples exhibited a gradual decline in ‘a’ values, consistent with the trend observed in Figure 1a. The ‘a’ value of sausages containing colorant lake at higher concentrations (Samples 10–12) consistently surpassed that of both the blank sample and the sodium nitrite sample on each measurement day. From 0 to 20 days, the ‘a’ value of the sausage with 0.26 g/kg MPs-CaCO_3_ lake (Sample 8) closely matched that of the samples with sodium nitrite (Sample 2) and even surpassed it during the latter part of the storage period. This indicates that 0.26 g/kg of MPs-CaCO_3_ lake achieves a similar red color and better color stability compared to 0.15 g/kg of sodium nitrite. Given the determined loading efficiency of MPs in the lake (46.66%), the actual amount of MPs in the sample with 0.26 g/kg lake was 0.12 g/kg. This is only half of the 0.24 g/kg MPs required to replace sodium nitrite, as calculated in Section 3.1.

The stability of the redness of Samples 8–12 against steaming treatment was evaluated, with the results shown in Figure 2b. Compared to samples containing the colorant lake, the Δa value of the blank sample (Sample 1) did not significantly differ from Samples 8, 9, and 10. However, it was noticeably lower than that of Samples 11 and 12. This contrasts with Figure 1b, where the Δa value of the blank sample was lower than all samples with added MPs. These findings confirm that the heat stability of MPs improves when processed into the colorant lake.

Figure 2c illustrates the change in Δa of the samples, reflecting the color stability against light exposure, during storage. The blank sample consistently exhibited the lowest Δa due to its initially low ‘a’ value (as shown in Figure 2a). For the sample with sodium nitrite (Sample 2) and those with MPs lake (Samples 8–12), their Δa values fluctuated but showed an overall increasing trend. The sample with 0.26 g/kg MPs-CaCO_3_ lake (Sample 8), capable of replacing the maximum amount of sodium nitrite as indicated in Figure 2a, consistently had lower Δa values than the sodium nitrite sample. These results confirm that at this concentration (0.26 g/kg), the colorant lake can substitute for sodium nitrite for dyeing and offers greater light stability. For the samples with the addition of lake, their lowest Δas were observed alternately in each of them during the first 14 days of the storage experiment. However, after 14 days, Sample 2 consistently maintained the lowest Δa value. Sample 11 demonstrated lower or comparable light stability to Sample 9, although it was less stable than Sample 10, despite containing more MPs.

Upon review of the analysis, it is evident that the experimental application of substituting sodium nitrite with lake in the production of smoked sausages has yielded promising results. The use of MPs-CaCO_3_ lake has demonstrated a remarkable capability to enhance both the heat and light stability of MPs. This lake formulation exhibited an impressive capacity to replace sodium nitrite in the coloration process of the sausages. Furthermore, the incorporation of colorant lake technology not only bolstered the stability of MPs but also significantly amplified their coloring efficacy in meat products. Notably, the same color intensity was achieved with a 50% reduction in the actual quantity of MPs applied. The experimental outcomes corroborate that the application of colorant lake technology offers dual benefits: it significantly reduces the required dosage of MPs while simultaneously augmenting their stability. This is particularly noteworthy in the context of smoked sausages, where color stability and safety are paramount concerns. These results suggest that the MPs-CaCO_3_ lake formulation could represent a promising alternative to traditional coloring methods in the meat processing industry, potentially leading to more stable, efficient, and cost-effective coloration processes.

### 3.3. Substituting Sodium Nitrite with Nisin

**Figure 3:**
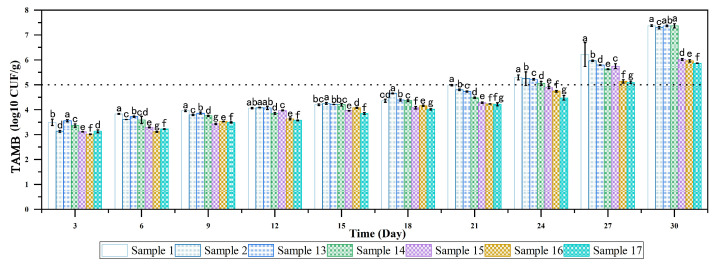
Variation of total aerobic mesophilic bacteria count in smoked sausages with different supplemental levels of nisin during storage. Different letters indicate statistically significant differences between the groups (*p* < 0.05).

Nisin was used to replace sodium nitrite to realize its antibacterial activity. The influence of nisin concentration on the TAMB of sausages during storage is shown in Figure 3.

In accordance with the Chinese National Standard for Cooked Meat Products (GB 2726-2016 [36]), the permissible maximum TAMB for smoked sausage is at 5 log10 CFU/g, as indicated by the dotted line in Figure 3. Figure 3 illustrates that across all experimental groups, the TAMB values progressively increased throughout the storage duration. During the initial 21 days, the TAMB values for all groups fluctuated between 3–5 log10 CFU/g, consistently remaining beneath the regulatory threshold. By the 24th day, the TAMB for the sample containing sodium nitrite, as well as those with nisin at concentrations below 0.2 g/kg, had surpassed the stipulated limit, signifying the onset of spoilage in the smoked sausages. Notably, the samples with nisin at 0.2 and 0.3 g/kg were found to be marginally above the threshold. On the 27th day, the TAMB for the samples with 0.2 and 0.3 g/kg nisin also crossed the cautionary line, while the addition of 0.4 and 0.5 g/kg nisin yielded the most effective preservation results. By the 30th day, all samples had exceeded the cautionary line.

The experimental findings indicate that an increased concentration of nisin correlates with an extended shelf life for the sausages. However, the preservation efficiency was identical for concentrations of 0.4 and 0.5 g/kg nisin, both maintaining the product for 27 days. Economic considerations suggest that a nisin concentration of 0.4 g/kg is the optimal choice for preservation.

Incorporating the results from the experiments substituting sodium nitrate with lake, it can be deduced that smoked sausages produced with 0.26 g/kg of lake and 0.4 g/kg of nisin can effectively replicate the coloration and antimicrobial properties of sausages containing 0.15 g/kg of sodium nitrate. However, further discussion of the bacteriostatic effect of nisin would be enhanced by conducting bacteriostatic experiments on *C. botulinum*, the Enterobacteriaceae family and *Salmonella*.

### 3.4. Physicochemical Analysis of Smoked Sausages

#### 3.4.1. Effect of MPs-CaCO_3_ Lake on the pH of Smoked Sausages

**Figure 4:**
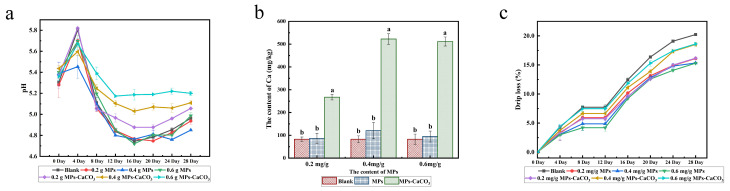
(**a**) The pH variation of the smoked sausage during storage; (**b**) Variation in calcium content of smoked sausages during storage (different letters indicate statistically significant differences between the groups (*p* < 0.05)); (**c**) Variation in the juice loss of smoked sausages during storage.

There exists an immense variety of sausages, each characterized by distinct pH levels dictated by the variations in raw materials and manufacturing methods. It is observed that within an optimal pH range, a higher pH value tends to improve both the color stability and the water-holding capacity of the sausages, as noted by Wang, et al. [37]. The homemade smoked sausages produced in this experiment typically exhibited a pH range of 5.3 to 6.2. Figure 4a delineates the pH fluctuations observed in samples with various pigment or lake incorporations throughout the storage duration. These pH values exhibited an initial increase, followed by a subsequent decline over the course of the study.

At the commencement of the experiment (day 0), the pH values of the samples were not significantly different (*p* > 0.05), ranging from 5.3 to 5.5. This indicates that the introduction of MPs and CaCO_3_ did not substantially alter the pH of the fresh sausages. During the initial four days of storage, it can be posited that the increase in pH at this time may be attributable to the presence of nitrogen compounds [38]. From day 4 to 16, a significant downward trend in pH values across all samples was noted, attributable to the decomposition of organic matter by microorganisms, which resulted in the production of acids and thus a decrease in pH. However, samples containing the colorant lake experienced a less pronounced pH decrease compared to the blank sample and those with MPs. This could be attributed to the buffering effect of CaCO_3_, which may influence microbial fermentation and act as an antacid. Post day 8, the pH values of samples with the colorant lake were significantly higher than those of the blank and pigment samples. After the 16th day, there was a gradual pH increase observed in the samples, and this may be attributed to the microbial breakdown of amino acids, which results in the production of alkaline compounds that accumulate over time [39].

#### 3.4.2. Effect of MPs-CaCO_3_ Lake on the Calcium Content in Smoked Sausage

Our results show that MPs-CaCO_3_ lake can replace MPs to dye meat products, enhancing their color. Additionally, the matrix of the colorant lake, calcium carbonate, serves as a calcium supplement. Therefore, the MPs-CaCO_3_ lake combines the functions of MPs and calcium carbonate, achieving the dual effect of coloring and providing calcium supplementation. Figure 4b demonstrates the effect of the same added amount of pigment and colorant lake on the content of elemental calcium in smoked sausages. The results showed that the calcium content in the samples with added colorant lake was significantly higher than that in the blank and pigment groups (*p* < 0.05), indicating that the MPs-CaCO_3_ lake was effective in calcium supplementation.

#### 3.4.3. Effect of MPs-CaCO_3_ Lake on Juice Loss of Smoked Sausage

The changes of juice loss of the samples with different additive amounts of pigment/colorant lake during the storage period are shown in Figure 4c. The juice loss of samples with the addition of different amounts of pigment/colorant lake showed an upward trend with a plateau from 8th to 12th day, and the increasing trend of the juice loss of the samples intensified after the 12th day of storage, which may be due to the fact that the samples reached the limit of rancidity and deterioration after that day [40]. During the measurement of juice loss, all other samples exhibited a lower juice loss rate than the blank samples, particularly in the later stages of storage. These results indicate that the juice loss rate of sausages can be reduced by incorporating both MPs and the colorant lake during the processing of smoking the sausage. Considering the composition of the MPs and the lakes, we can infer that the presence of MPs contributed to the improved water-holding capacity of the smoked sausages. However, the effectiveness of MPs and lake in reducing juice loss from sausage differed. By the end of the experiment (day 28), sausages with the addition of MPs at all concentration levels exhibited the lowest juice loss rate, around 12.5%. In contrast, only the sausage with the addition of 0.2 g of lake achieved a similarly low juice loss, while higher amounts of lake resulted in increased juice loss, reaching about 17%. These findings confirmed that higher concentrations of lake have an adverse effect on the water-holding capacity of the sausage. This may be due to the presence of large amounts of calcium carbonate, which cause an increase in the pH of the sausage, which in turn leads to protein distortion. This phenomenon reduces the ability of proteins to bind water, thereby facilitating water loss from the sausage.

### 3.5. Analysis of Flavor Compounds in Smoked Sausages

#### 3.5.1. Volatile Flavor Compounds in Sausages

The volatiles identified in the sausages in the blank group, pigment group, and lake groups are shown in Table 2. A total of 32 volatile compounds were identified in the blank sample, including 12 alkanes, 5 benzenes, 4 alcohols, 4 ketones, 3 phenols, 3 esters, 1 alkene; a total of 30 volatile compounds were identified in the pigment group sample, including 12 alkanes, 6 benzenes, 4 esters, 3 phenols, 3 ketones, 1 aldehyde, 1 furan; and a total of 30 volatile compounds were identified in the lake group sausages, including 12 alkanes, 6 benzenes, 4 esters, 3 phenols, 3 ketones, 1 aldehyde, 1 furan. The main volatile compounds in the blank sausage were 3-hydroxy-2-butanone and (S)-isopropyl lactate; in the pigment group, they turned out to be triacetaldehyde, (S)-isopropyl lactate, and 3-hydroxy-2-butanone; and in the lake group, they were triacetaldehyde, 3-hydroxy-2-butanone, and (S)-isopropyl lactate. For the identified volatile compounds, the relative abundance of each compound was calculated. The most abundant volatile compounds were ketones and esters in the blank group; alkanes, esters, and ketones in the pigment group; and ketones and esters in the lake group, indicating the addition of lake did not change the basic composition of the volatile flavor compounds in sausages.

Esters, primarily formed from the esterification of carboxylic acids and alcohols [41], were prevalent in the blank, pigment, and lake groups. The most abundant ester detected was (S)-isopropyl lactate. Additionally, small amounts of ethyl esters, such as ethyl acetate and vinyl acetate, were identified. These compounds are important for flavor formation in smoked sausages, with ethyl acetate contributing a fruity aroma.

Aldehydes are produced mainly through lipid oxidation and protein hydrolysis, having a low threshold and broad flavor presentation, thus contributing more prominently to the overall flavor of lightly fermented sausages, and the effect of low-carbon aldehydes on the flavor of sausages was particularly significant [42]. Usually, C3 and C4 aldehydes have a strong irritating odor; C5~C9 aldehydes have oily-wax and greasy flavor; and C10~C12 aldehydes have lemony and orange peel flavors. Paraldehyde was high in the pigment group and lake group, but it was not found in the blank group. The hydrolysis of protein produces methionine, which decomposes to form acetaldehyde [43]. During the heating process, acetaldehyde undergoes a polymerization reaction to form paraldehyde, and the results suggest MPs facilitate this reaction.

Ketones are primarily produced through amino acid catabolism or carbohydrate metabolism [44]. 3-Hydroxy-2-butanone, a common methyl ketone found in sausages, was present at high levels in all three groups of sausages. This compound is associated with fat oxidation and contributes significantly to the fatty aroma of sausages after smoking [45].

Alcohols originate from various sources, with carbohydrate metabolism being the primary contributor. Among all the alcohols, (2R,3R)-(-)-2,3-butanediol was detected in the blank group but not in the pigment and lake groups. This suggests that the MPs may have exerted an inhibitory effect on microbial reproduction, resulting in the absence of significant alcohol production in those groups [46].

**Figure 5:**
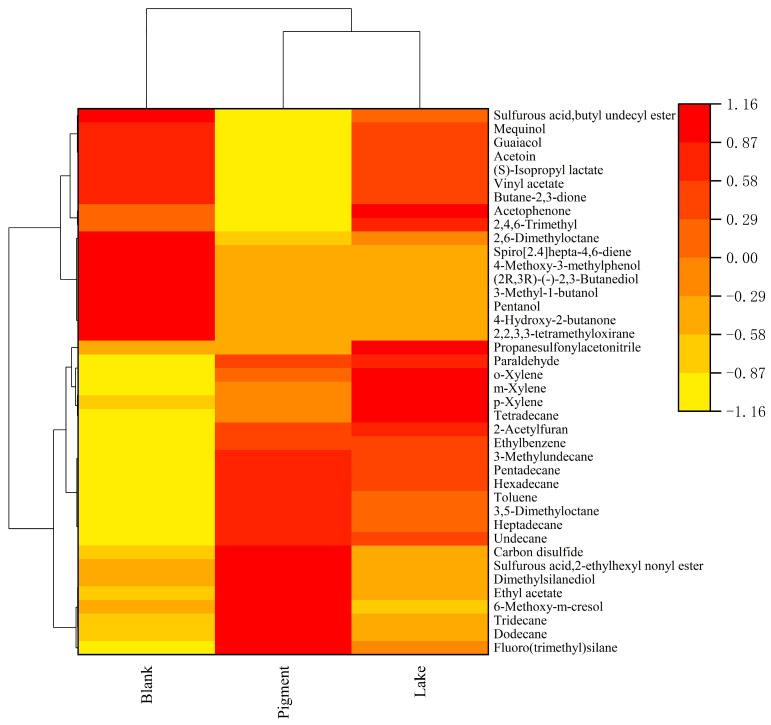
Heat map and clustering results of volatile compounds present in the three sets of samples.

From the above analysis, it can be seen that the types of volatile flavor substances are similar between the sausages in the pigment group and the sausages in the lake group, indicating that the effect of the colorant lake on the flavor of sausages can reach the same level as that of the pigments alone. And the addition of MPs may have reduced microbial growth and multiplication in smoked sausages to some extent. The distribution of volatile compounds in the sausages from the three groups were visualized using a heat map, as shown in Figure 5, where each row represents a distinct volatile compound. The more the color approaches red, the higher the concentration of the substance. Based on Figure 5 and Table 2, it is evident that the sausages from the pigment group and the lake group exhibited similar compositions of volatile flavor substances, suggesting that the presence of MPs significantly influenced the flavor profile of the sausages.

#### 3.5.2. Odor Analysis of Sausages Using Electronic Nose

The PEN3 electronic nose is used for odor fingerprinting data collection through an array system with 10 metal oxide sensors [47]. Each sensor in the sensor arrays is sensitive to a particular type of substance when the gas passes through the array, and they can correspondingly produce a characteristic response. This response value is expressed as the ratio of the conductivity of the sample gas (G) to the conductivity of the background air (G_0_). The characteristic odor fingerprint of this substance is formed by collecting the ratios of the conductivity of 10 sensors.

**Figure 6:**
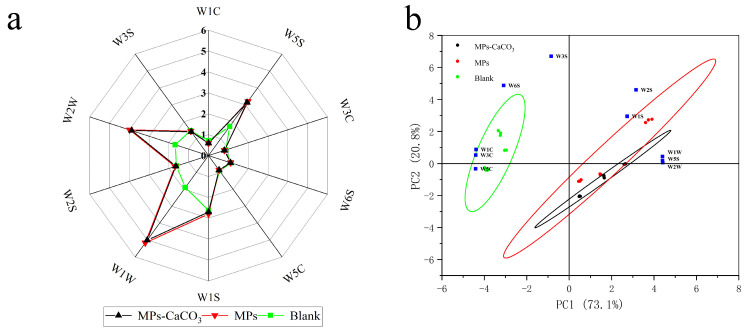
(**a**) Radar chart of the e-nose sensor response signals; (**b**) biplot of PCA analysis of the e-nose data.

The ten sensors of the electronic nose are W1C (aromatic constituents, benzene), W5S (highly sensitive and sensitive to nitrogen oxides), W3C (sensitive aroma ammonia), W6S (mainly selective for hydrides), W5C (short-chain alkane aromatic component), W1S (sensitive to methyl), W1W (sensitive to sulfides), W2S (sensitive to alcohol, aldehydes and component), W2W (aromatics ingredients, sensitive to organic sulfides), and W3S (sensitive to long-chain alkanes) [48]. As shown in Figure 6a, the sensors of the electronic nose showed an obvious response to the odor compounds in all three groups of sausage samples, with W1C, W3C and W5C and W6S having the lower responses (about one or less) in each group of samples and W1W, W1S, W2W, W2S, W3S and W5S having much larger responds. This is similar to the e-nose analysis results obtained from sausages smoked with different woodchips by Yin et al. [48]. As shown in Figure 6b, PC1 is positively correlated with W1S, W2S, W5S, W1W and W2W, and negatively correlated with W3S, W6S, W1C, W3C and W5C; PC2 is positively correlated with W1S, W2S, W3S, W5S, W6S, W1W, W2W, W1C and W3C, and negatively correlated with W5C. The compounds with low responses in the e-nose analysis were predominantly those with low relative concentrations or undetectable in the volatile flavor analysis (Table 2), suggesting a notable correlation between the two methods. For the pigment group and lake group, their lines in the radar chart almost overlapped, suggesting high similarity between the smell of the two kinds of samples. Additionally, the blank group (green line) was obviously different from that of the pigment group and lake group, confirming a significant discrepancy in smell caused by the addition of MPs. This difference is primarily reflected in the readings from three sensors: W1W, W2W, and W5S, which are responsible for detecting nitrogen oxides, sulfur compounds, and aromatic and organosulfur compounds, respectively. This indicates that the levels of nitrogen oxides, sulfur compounds, and aromatic and organosulfur compounds were higher in the sausages from the pigment and lake groups compared to those from the blank group, which is consistent with the results presented in Table 2. Possible explanations for this include the bioactive properties of MPs, which may interact with proteins to generate a greater number of nitrogen- and sulfur-containing flavor compounds by influencing the Maillard reaction and Strecker degradation [49]. Additionally, the antioxidant property of MPs can also slow down the oxidation of fats, preserving more flavor precursors that can convert into nitrogen- and sulfur-containing flavor compounds. Lastly, the pigment might affect enzymatic reactions in the sausage, particularly those involving sulfur-containing amino acids, leading to an increase in these flavor compounds [50].

From the results of PCA analysis, as shown in Figure 6b, the sum of the contribution of the first and second principal components is more than 99%, with the contribution of the first principal component accounting for 20.8% and that of the second principal component accounting for 73.1%. The distribution of the samples in the graph reveals a significant overlap between the pigment group and the lake group among the three types of sausages, indicating that the odor substances in both groups are similar. This suggests that there is no substantial difference in the odor profiles of sausages containing pigments and those with lake, confirming that lake could serve as a substitute for pigments. However, the distinction between these two groups and the blank group is quite pronounced.

#### 3.5.3. Taste Analysis of Sausages Using Electronic Tongue

The electronic tongue is an effective means for analyzing gustatory substances with an array of sensors that mimic human taste perception. We used the SA402B electronic tongue, employing an analytical system containing a total of five sensors and three standard electrodes for C00 (bitterness sensor), AE1 (astringency sensor), CA0 (sourness sensor), CT0 (saltiness sensor), and AAE (umami sensor) [51].

**Figure 7:**
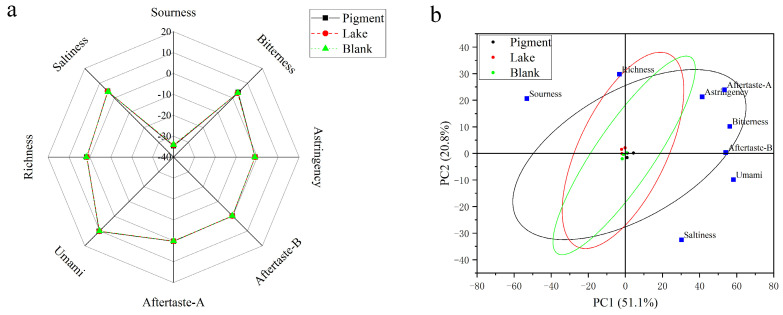
(**a**) Radar chart of the e-tongue sensor response signals; (**b**) biplot of PCA analysis of the e-tongue data.

The pigment group, lake group, and blank group are shown in Figure 7a. The differences among the lake sausages, pigment sausages and blank sausages were not significant (*p* > 0.05) in terms of umami, saltiness, bitter aftertaste (aftertaste-B), astringent aftertaste (aftertaste-A) or richness (sustainably perceived umami). Whereas, in terms of astringency, sourness and bitterness, the blank sample was significantly lower (*p* < 0.05) than sausages from the lake group and pigment group.

Based on the PCA analysis, the two principal components accounted for a total of 71.9% of the variance, with PC1 contributing 51.1% and PC2 contributing 20.8%. These components effectively capture the primary information regarding the taste profiles of the lake group, pigment group, and blank group. As shown in Figure 7b, PC1 was positively correlated with aftertaste-A, astringency, bitterness, aftertaste-B, umami and saltiness, and negatively correlated with sourness and richness; PC2 was positively correlated with aftertaste-A, astringency, bitterness, aftertaste-B, sourness and richness, and negatively correlated with umami and saltiness. The points representing the three groups of sausages exhibit a tendency to cluster in the two-dimensional space shown in Figure 7b, suggesting that the lake group, pigment group, and blank group are remarkably similar in terms of taste. This indicates that the addition of colorant lake does not noticeably alter the flavor profile of the sausages [52].

## 4. Conclusions

In this study, we investigated the effects of using MPs-CaCO_3_ lake and nisin as substitutes for nitrite in the production of smoked sausages through sensory analysis, physicochemical analysis, and flavor analysis. The results indicated that a coloring effect similar to that of nitrite could be achieved by adding the colorant lake at a concentration of 0.26 g/kg. Additionally, nisin demonstrated potential as a nitrite substitute due to its antibacterial and antiseptic properties when added at 0.4 g/kg in the sausage formulation. The incorporation of the colorant lake also improved the pH value, reduced juice loss, and increased the calcium content of the sausages during storage. Sensory evaluations revealed no significant differences in ratings for fragrance or taste throughout the storage period among the three types of sausages. Both the lake and pigment groups consistently received the highest scores for color throughout the experiment, with the lake group showing greater acceptability toward the end of the storage period. GC-MS analysis identified the absence of butylene glycol and the presence of paraldehyde in both the lake and pigment groups, in contrast to the blank group. Electronic nose assessments found no significant differences in the odor profiles between the pigment and lake groups; however, a notable difference was observed when compared to the blank group. Electronic tongue evaluations indicated that the flavor profiles of the lake, pigment, and blank groups were remarkably similar. This study demonstrated the feasibility of using MPs-CaCO_3_ lake and nisin in combination as substitutes for nitrite in smoked sausage production. Furthermore, it highlights several advantages of using lake as a pigment alternative. 

The findings of this research provide innovative insights for enhancing pigment efficiency and reducing reliance on traditional additives in the food industry. The present study provides a comprehensive analysis of the application of MPs-CaCO_3_ lake in sausages. However, this study has not yet addressed the critical aspect of toxicological evaluation, which is a mandatory component of assessing its safety as a food additive. Therefore, future studies must carry out further systematic toxicological analyses, including acute toxicity, genotoxicity, and chronic toxicity tests, to ensure the safety of this pigment in food applications. Further research is needed to explore the broader applications and potential limitations of these pigments in the food industry.

## Figures and Tables

**Table 1:** Formulations for sausages produced in the substitution experiment (Section 2.3.1, Section 2.3.2 and Section 2.3.3).

Samples	Nisin (g/kg)	Sodium Nitrite (g/kg)	MPs (g/kg)	MPs-CaCO_3_ Lake (g/kg)
1	0	0	0	0
2	0	0.15	0	0
3	0.5	0	0.4	0
4	0.5	0	0.6	0
5	0.5	0	0.8	0
6	0.5	0	1.3	0
7	0.5	0	1.6	0
8	0.5	0	0	0.26
9	0.5	0	0	0.36
10	0.5	0	0	0.51
11	0.5	0	0	0.67
12	0.5	0	0	0.77
13	0.1	0	0	0.26
14	0.2	0	0	0.26
15	0.3	0	0	0.26
16	0.4	0	0	0.26
17	0.5	0	0	0.26

**Table 2:** Volatile compounds from sausages.

Volatile Compounds	CAS Number	Blank Group Relative Content (%)	Pigment Group Relative Content (%)	Lake Group Relative Content (%)
**Alkanes**				
Fluoro(trimethyl)silane	420-56-4	-	2.690 ^a^ ± 1.525	0.943 ^b^ ± 0.304
Undecane	1120-21-4	1.906 ^c^ ± 0.354	4.746 ^a^ ± 0.421	4.023 ^b^ ± 0.411
Dodecane	112-40-3	3.210 ^c^ ± 0.616	6.136 ^a^ ± 0.530	3.743 ^b^ ± 1.529
Tridecane	629-50-5	1.303 ^c^ ± 0.262	3.123 ^a^ ± 0.979	1.486 ^b^ ± 0.313
Hexadecane	544-76-3	2.080 ^c^ ± 0.400	3.816 ^a^ ± 0.888	3.626 ^b^ ± 1.024
Tetradecane	629-59-4	0.683 ^c^ ± 0.066	0.703 ^b^ ± 0.203	0.723 ^a^ ± 0.132
Pentadecane	629-62-9	0.890 ^c^ ± 0.246	1.673 ^a^ ± 1.229	1.593 ^b^ ± 0.716
Heptadecane	629-78-7	0.683 ^c^ ± 0.066	1.040 ^a^ ± 0.347	0.933 ^b^ ± 0.340
2,2,3,3-tetramethyloxirane	5076-20-0	1.233 ± 0.023	-	-
2,4,6-Trimethyl octane	62016-37-9	1.530 ^b^ ± 0.096	1.216 ^c^ ± 0.277	1.676 ^a^ ± 0.152
2,6-Dimethyloctane	2051-30-1	0.670 ^a^ ± 0.030	0.490 ^c^ ± 0.182	0.540 ^b^ ± 0.119
3-Methylundecane	1002-43-3	0.423 ^b^ ± 0.077	0.483 ^a^ ± 0.059	0.480 ^a^ ± 0.104
3,5-Dimethyloctane	15869-93-9	1.906 ^c^ ± 0.354	2.943 ^a^ ± 0.503	2.630 ^b^ ± 0.516
**Benzenes**				
p-Xylene	106-42-3	5.706 ^c^ ± 0.200	7.313 ^b^ ± 1.252	10.726 ^a^ ± 0.892
Toluene	108-88-3	0.383 ^c^ ± 0.029	0.613 ^a^ ± 0.147	0.543 ^b^ ± 0.098
m-Xylene	108-38-3	6.886 ^c^ ± 0.237	8.936 ^b^ ± 1.657	12.350 ^a^ ± 1.077
o-Xylene	95-47-6	2.716 ^c^ ± 0.726	8.156 ^b^ ± 1.095	11.146 ^a^ ± 2.115
Ethylbenzene	100-41-4	-	1.623 ^a^ ± 0.407	1.623 ^a^ ± 0.185
6-Methoxy-m-cresol	1195-09-1	0.460 ^b^ ± 0.064	1.783 ^a^ ± 0.140	0.366 ^c^ ± 0.056
**Ketones**				
Acetophenone	98-86-2	1.343 ^b^ ± 0.026	1.166 ^c^ ± 0.162	1.503 ^a^ ± 0.142
Butane-2,3-dione	431-03-8	1.700 ^a^ ± 0.064	0.890 ^c^ ± 0.381	1.583 ^b^ ± 0.047
Acetoin	513-86-0	48.730 ^a^ ± 4.113	34.450 ^c^ ± 6.781	46.003 ^b^ ± 1.888
4-Hydroxy-2-butanone	590-90-9	0.533 ± 0.098	-	-
**Esters**				
Ethyl acetate	141-78-6	0.533 ^b^ ± 0.098	1.040 ^a^ ± 0.347	0.540 ^b^ ± 0.119
Vinyl acetate	108-05-4	1.700 ^a^ ± 0.064	0.890 ^c^ ± 0.39	1.583 ^b^ ± 0.047
Dimethylsilanediol	1066-42-8	-	2.690 ^a^ ± 1.525	2.463 ^b^ ± 1.473
(S)-Isopropyl lactate	63697-00-7	48.730a ± 4.113	32.150 ^c^ ± 6.546	46.003 ^b^ ± 1.888
**Alcohols**				
Pentanol	71-41-0	0.626 ± 0.175	-	**-**
3-Methyl-1-butanol	123-51-3	0.626 ± 0.175	-	**-**
(2R,3R)-(-)-2,3-Butanediol	513-85-9	3.216 ± 0.649	-	**-**
**Phenols**				
Guaiacol	90-5-1	1.486 ^a^ ± 0.104	1.080 ^c^ ± 0.207	1.376 ^b^ ± 0.182
Mequinol	150-76-5	1.486 ^a^ ± 0.104	1.080 ^c^ ± 0.207	1.376 ^b^ ± 0.182
4-Methoxy-3-methylphenol	14786-82-4	0.460 ^a^ ± 0.064	0.366 ^b^ ± 0.056	0.366 ^b^ ± 0.056
**Sulfide**				
Carbon disulfide	75-15-0	0.390 ^c^ ± 0.032	0.650 ^a^ ± 0.086	0.400 ^b^ ± 0.05
Propanesulfonylacetonitrile	175137-61-8	-	-	1.825 ± 0.047
Sulfurous acid, butyl undecyl ester	0-00-0	0.463 ± 0.170	-	0.290 ± 0.046
Sulfurous acid,2-ethylhexyl nonyl ester	0-00-0	-	0.110 ± 0.021	-
**Others**				
Paraldehyde	123-63-7	-	34.450 ^b^ ± 6.781	46.003 ^a^ ± 1.888
2-Acetylfuran	1192-62-7	-	0.410 ^b^ ± 0.106	0.433 ^a^ ± 0.058
Spiro[2.4]hepta-4,6-diene	765-46-8	0.383 ± 0.029	-	-

Different letters indicate statistically significant differences between the groups (*p* < 0.05). The presence of a short bar in the table signifies that the corresponding value was not detected.

## Data Availability

The original contributions presented in the study are included in the article, further inquiries can be directed to the corresponding author.
